# Pre-excitation syndrome: ablating the pathway

**DOI:** 10.3402/jchimp.v2i2.18912

**Published:** 2012-07-16

**Authors:** Marc Mugmon

**Affiliations:** Department of Medicine, Union Memorial Hospital, Baltimore, MD, USA

## Abstract

Disappearance of a delta wave in a patient with pre-excitation is demonstrated following successful ablation.

A 52 year-old white male suddenly developed a sensation of chest fullness followed by severe lightheadedness and a feeling as if he were about to pass out. After several minutes these symptoms were followed by several minutes of severe palpitations, after which time he felt fine.

He had had palpitations for many years and at the age of 18 was told that he had Wolff–Parkinson–White-Syndrome. However, he had not been following up with doctors on a regular basis. Cardiac catheterization performed for an evaluation of chest pain at age 43 revealed no significant coronary artery disease and normal systolic function and pressures. He denied prior episodes of syncope or dizziness, but palpitations had been relatively frequent his entire life. His only medication was daily aspirin and he denied any allergies.

Past medical history was otherwise unremarkable. Family history was negative for known cardiac problems. Social history was characterized by a former smoking history, which he had stopped 12 years before. He drank several beers daily and denied drug use. He was very active physically and worked as a contractor.

Physical examination was normal except for an initial blood pressure of 172/91. Laboratory studies were normal except for TSH of 5.37 (normal 0.400–4.0) and a positive urine screen for cannabinoids.

Electrocardiogram on admission (Fig. 1) revealed what appeared to be a short PR interval and a wide QRS, with slurring of the initial portion of the QRS consistent with a delta wave, indicating ventricular pre-excitation, compatible the previous diagnosis of Wolff–Parkinson–White Syndrome. The characteristics of the delta wave suggested the presence of a right posterior septal accessory pathway.

**Fig. 1:**
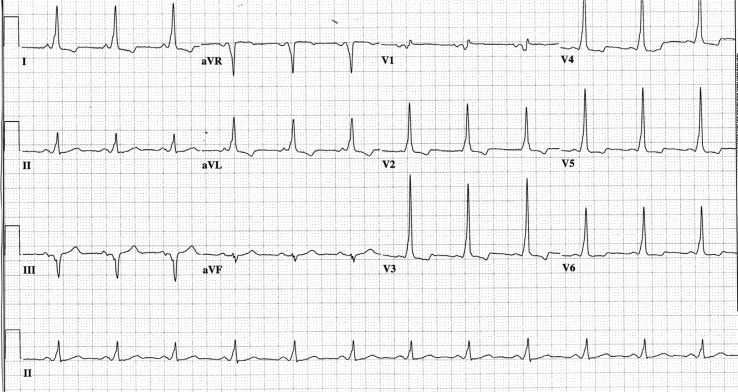
ECG showing short PR, delta waves and wide QRS duration.

Electrophysiologic testing was performed. Antegrade conduction across the accessory pathway was demonstrated and improved with isoproterenol. However, no reentrant tachycardia could be induced during the procedure, despite aggressive stimulation and isoproterenol infusion, and no retrograde conduction over the pathway could be demonstrated. Because of the possibility that he may have had an antidromic tachycardia (meaning conduction down the accessory pathway and back through the AV node), ablation of the accessory pathway was performed.

The electrocardiogram following the procedure (Fig. 2) reveals a normal tracing, with the delta wave no longer present and the QRS duration being normal, indicating resolution of ventricular pre-excitation due to ablation of the accessory pathway.

**Fig. 2:**
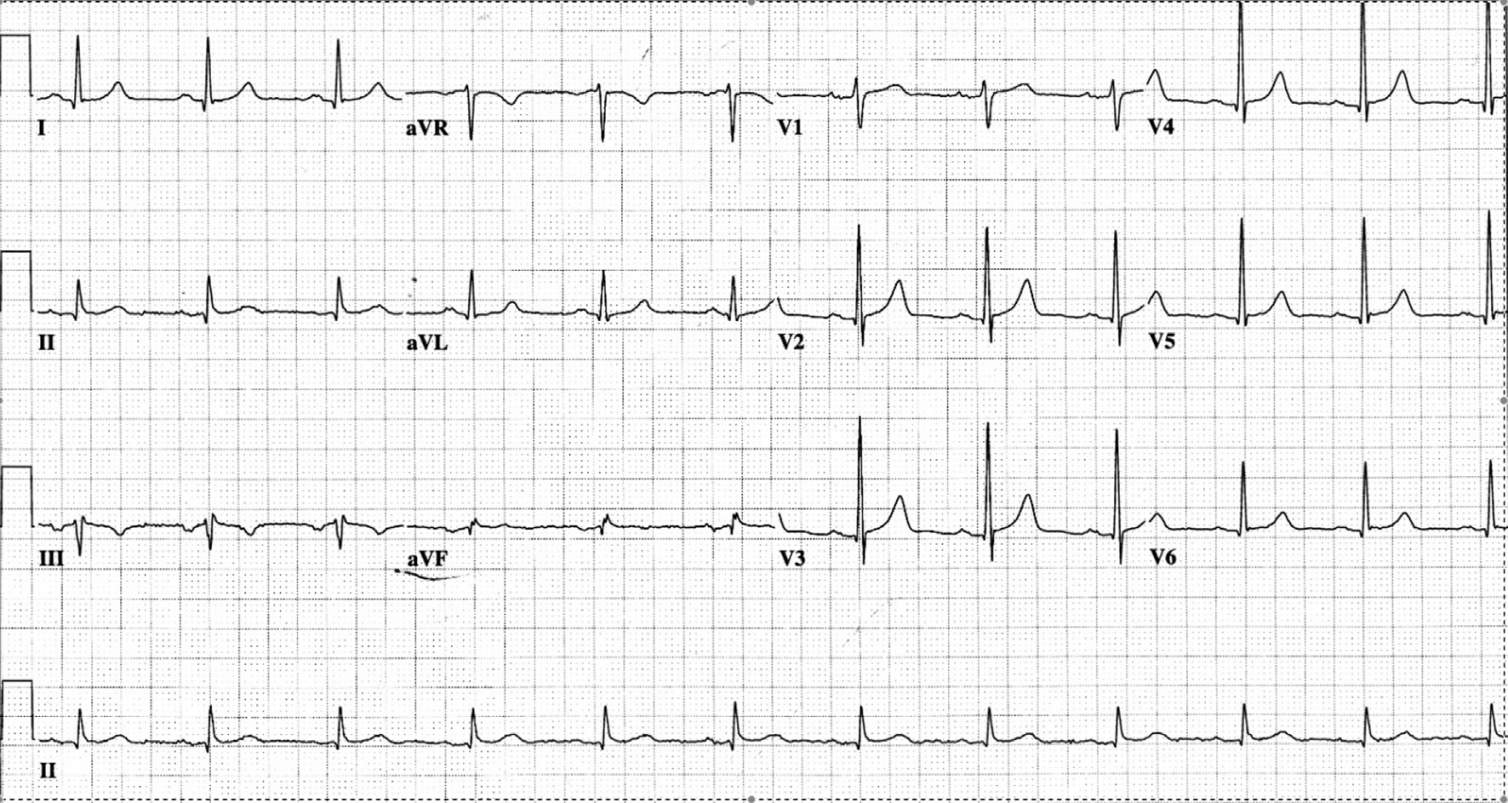
ECG after ablation, demonstrating normal PR, absence of delta wave, and normal QRS duration.

Three months after the procedure the patient remains free of palpitations, and the tracing remains normal.

This patient's tracings demonstrate the return to normal of the surface electrocardiogram after successful ablation of an accessory pathway. Many patients with pre-excitation may have intermittent accessory pathway conduction. Pre-excitation many occur only at lower rates and may disappear at higher rates, or may be affected by vagal influences. However, all of this patient's prior electrocardiograms revealed pre-excitation, and only after ablation did the delta wave disappear.

Readers who are interested in the many facets of this condition are referred to the original 1930 article describing these patients (1), as well as the many review articles available.
